# Higher serum CCL17 may be a promising predictor of acute exacerbations in chronic hypersensitivity pneumonitis

**DOI:** 10.1186/1465-9921-14-57

**Published:** 2013-05-25

**Authors:** Yasunari Miyazaki, Koji Unoura, Tomoya Tateishi, Takumi Akashi, Tamiko Takemura, Makoto Tomita, Naohiko Inase, Yasuyuki Yoshizawa

**Affiliations:** 1 Departments of Respiratory Medicine, Tokyo Medical and Dental University, Tokyo, Japan; 2 Departments of Pathology, Tokyo Medical and Dental University, Tokyo, Japan; 3 Department of Pathology, Japanese Red Cross Medical Center, Tokyo, Japan; 4 Clinical Research Center, Tokyo Medical and Dental University, Tokyo, Japan

**Keywords:** Chronic hypersensitivity pneumonitis, Acute exacerbations, CCL17/thymus- and activation-regulated chemokine, C-C chemokine receptor 4

## Abstract

**Background:**

Recent research has suggested that the Th1 and Th2 chemokine/cytokine axis contributes to the development of chronic hypersensitivity pneumonitis (HP). Acute exacerbations (AE) are significant factors in the prognosis of chronic HP. Little is known, however, about these biomarkers in association with AE in chronic HP patients.

**Methods:**

Fifty-six patients with chronic HP were evaluated, including 14 patients during episodes of AE. Th1 mediators (C-X-C chemokine ligand [CXCL]10 and interferon [IFN]-γ), Th2 mediators (C-C chemokine ligand [CCL]17, interleukin-4, and interleukin-13), and pro-fibrotic mediator (transforming growth factor [TGF]-β) were measured to evaluate the mediators as predictors of AE. C-C chemokine receptor (CCR)4 (receptor for CCL17)-positive lymphocytes were quantified in lung specimens.

**Results:**

Serum CCL17 levels at baseline independently predicted the first episode of AE (HR, 72.0; 95% CI, 5.03-1030.23; p = 0.002). AE was significantly more frequent in the higher-CCL17 group (≥285 pg/ml) than in the lower-CCL17 group (<285 pg/ml) (log-rank test, p = 0.0006; 1-year incidence: higher CCL17 vs. lower CCL17, 14.3% vs. 0.0%). Serum CCL17 levels and CCR4-positive cells during episodes of AE were increased from the baseline (p = 0.01 and 0.031).

**Conclusions:**

Higher serum concentrations of CCL17 at baseline may be predictive of AE in patients with chronic HP, and CCL17 may contribute to the pathology of AE by inducing the accumulation of CCR4-positive lymphocytes in the lungs.

## Background

Acute exacerbations (AE) have been recognized as a major complication in the prognosis of idiopathic pulmonary fibrosis (IPF) over the last decade [1-4]. AE can also occur during the course of chronic hypersensitivity pneumonitis (HP), and when it does it exhibits a clinical course similar to that in IPF [5-7]. Our group has recently shown that patients with a usual interstitial pneumonia (UIP) pattern have a poor prognosis in chronic HP [7].

Earlier results from our group suggested that the Th2-predominant immune response may play an important role in the development of the UIP pattern in chronic HP [8]. Barrera *et al*. observed a skewing of Th2 activity, a likely cause of fibrotic processes, in patients with chronic HP [9]. Th1 and Th2 cells are thought to play a pivotal role in fibrogenesis [10]. Investigators can measure the Th1/Th2 balance by evaluating chemokine receptors on T cells, using C-X-C chemokine receptor (CXCR)3 as a marker for Th1 cells and C-C chemokine receptor (CCR)4 as a marker for Th2 cells [11]. In a bleomycin mouse model, C-C chemokine ligand (CCL)17, the ligand of CCR4, contributed to the development of pulmonary fibrosis [12]. In contrast, CXCR3 and C-X-C chemokine ligand (CXCL)10 played non-redundant roles in attenuating fibrosis [13,14].

Biological markers of pulmonary fibrosis are important in a clinical setting. Prasse *et al.* recently showed that baseline levels of serum CCL18 are strong predictors of death in IPF patients [15]. They concluded that Th2-type chemokines were significant biomarkers of pulmonary fibrosis, as CCL18 is produced by M2 macrophages activated by Th2 cytokines. Similarly, previous human and animal studies of our own suggest that a Th2-skewed immune response has a crucial role in the clinical course of chronic HP [8,16].

Looking at this evidence, we hypothesized that the Th1/Th2 balance varies throughout the clinical course of chronic HP and that an immune response skewed toward Th2 is predictive of AE. In the present study we evaluated the utility of Th1 mediators (interferon [IFN]-γ and CXCL10), Th2 mediators (CCL17, interleukin [IL]-4, and IL-13), and pro-fibrotic mediator (transforming growth factor [TGF]-β) as predictors of AE in chronic HP. CCR4-positive lymphocytes with reference to the pathogenesis of chronic HP with AE were also assessed. We showed that the baseline serum level of CCL17 was the first predictor of the incidence of acute exacerbations in chronic hypersensitivity pneumonitis.

## Methods

### Patient selection

Patients who fulfilled all of the criteria for chronic HP at Tokyo Medical and Dental University Hospital from 1993 to 2009 were recruited. Among them, 56 patients with chronic bird fanciers’ lung (BFL), a type of HP, were enrolled as subjects. Some of the patients died and were autopsied. We reviewed the medical records and BAL profiles, and collected blood and BAL samples from all patients as baseline data at first admission. All of the patients underwent surgical lung biopsies. Thirty-seven patients tested positive in an inhalation provocation test and the other 19 tested positive in an environmental provocation test [17]. Most of the subjects had been included in previous studies by our group. No patients had medical histories of atopic dermatitis or bronchial asthma. Eleven healthy volunteers (HV) with no atopic medical history were enrolled as controls. The study conformed to the Declaration of Helsinki and was approved by the internal review board of Tokyo Medical and Dental University Hospital (approval number: 514).

### Criteria

The diagnostic criteria for chronic BFL included the following: 1) a history of avian contact, 2) antibodies and/or lymphocyte proliferation against avian antigens, 3) reproduction of the symptoms of HP by an environmental provocation or a laboratory-controlled inhalation of avian antigens [18], 4) progressive deterioration of a restrictive impairment on pulmonary function for at least 1 year, 5) respiratory symptoms related to HP for at least 6 months, and either 6) evidence of pulmonary fibrosis with or without granulomas on histopathological analysis or 7) honeycombing on computed tomography (CT) scans [19,20].

The criteria of Kondoh *et al*. were used to define AE [21]. 1) exacerbation of dyspnea for a duration of no more than 1 month, 2) hypoxemia with an arterial oxygen/fraction of inspired oxygen ratio (PaO_2_/FIO_2_) of < 225, 3) newly developed pulmonary infiltrates on chest radiography and 4) the absence of apparent infection or heart disease.

### Imaging

High- resolution computed tomography (HRCT) was performed with standard technical parameters at the time of diagnosis. Three pulmonary specialists (Y.M., K.U., T.T.) analyzed HRCT findings without knowledge of the patients’ clinical course and the Kazernooni score for fibrosis pattern and ground glass pattern was evaluated [22].

### Bronchoalveolar lavage (BAL)

We performed BAL as previously described, using three 50 ml aliquots of sterile 0.9% saline solution [8]. The cellular composition of the BAL was determined with a cytospin smear with a Wright stain and counting 200 cells. Lymphocyte phenotyping was performed by flow cytometry with monoclonal antibodies for CD3, CD4 and CD8.

### Measurements of CXCL10, CCL17, IFN-γ, IL-4, IL-13, and TGF-β

Levels of CXCL10, CCL17, IFN-γ, IL-4, IL-13 and TGF-β were measured in diluted (1:5) serum samples at either baseline (AE: n = 14, NAE: n = 42) or at indicated times and in undiluted BALF samples at either baseline (AE: n = 11, NAE: n = 37) or at indicated times. TGF-β1 was measured after converting latent TGF-β1 to active TGF-β1 by acidification. Although measuring active TGF-β1 is a better method to assess its physiological effect, we measured total TGF-β1 to evaluate this mediator as a predictor of AE because we could not detect the active form of TGF-β1 in most of the samples in this study. All measurements were performed using commercial immunoassays (Duoset, R & D Systems, Minneapolis, MN, USA), according to the manufacturer’s instructions.

### Histopathological evaluation

Lung tissues were obtained by video-assisted thoracoscopic surgery. Histological sections of biopsy materials were stained with hematoxylin-eosin and Elastica van Gieson. Histological examinations were interpreted by two pulmonary pathology specialists (T.T. and T.A.) who were blinded to the patient’s clinical information. Histologic patterns of lung specimens were classified according to the ATS/ERS international consensus classification as having a UIP pattern, a NSIP pattern, or an organizing pneumonia (OP) pattern, based on the quality of fibrotic changes, which included loose and dense fibrosis and the temporal appearance [1,23,24]. Patients with NSIP were subdivided into two groups: those with a cellular NSIP (cNSIP) pattern and those with a fibrotic NSIP (fNSIP) pattern [17].

### Immunohistochemical analysis

Paraffin sections (4-μm thick) from surgical lung biopsies and autopsies were prepared for immunohistochemistry. The following were used as primary monoclonal antibodies: CCL17 (R & D Systems), CCR4 (courtesy of Kyowahakko, Tokyo, Japan) and CD3 (DAKO Japan, Tokyo, Japan). Immunoperoxidase staining was performed as previously described [8]. For immunofluorescent staining, we used Alexa 488- or Alexa 546-conjugated streptavidin (Molecular Probes, Eugene, OR, US) instead of the ABC reagent. Fluorescent staining was analyzed using a FluoView 500 confocal microscope (Olympus, Melville, NY) at the Tokyo Medical and Dental University Imaging Facility.

### Semiquantification of CCR4-positive cells

Positive stains for infiltrating cells by anti-CCR4 immunohistochemistry were separately counted for fibrosing areas and for lymphoid clusters from surgical lung specimens and autopsy lung specimens, as previously described [8]. Positive cells were counted from 20 randomly selected high-power (400x) fields (HPFs) from fibrosing areas and from 5 HPFs from lymphoid clusters. Immunohistochemistry for CD3 was also performed on each serial section of the CCR4-stained sections to detect all T lymphocytes in the same fields and to enumerate the percentage of CCR4-positive cells among the T lymphocytes. The CCR4 results were expressed as the percentage of CD3 cells in the same field.

### Statistical analysis

The data were analyzed using GraphPad Prism version 5.0d (GraphPad Software Inc., San Diego, CA, US) and the R statistical software (http://www.r-project.org/). Values were expressed as medians and ranges, and a statistical significance level of 0.05 was used. The AE and NAE groups, or baseline and AE episodes were compared using the Mann-Whitney’s U test. Comparisons between groups were performed using χ^2^ or Fisher’s exact tests for categorical variables. The percentages of CCR4-positive cells at baseline and during an AE episode were compared using the Wilcoxon matched-pairs signed-rank test. In a receiver operating characteristic (ROC) analysis using the first episode of AE as an endpoint, the optimal cut-off point for serum CCL17 concentrations was defined as the point closest to the upper left corner of the ROC curve. Cox proportional hazard models were used to find the relationship between the first episode of AE and putative prognostic variables at baseline after adjustment for age, gender, smoking status, and the predicted vital capacity (VC) percent.

## Results

### Clinical features of chronic HP with AE

The baseline characteristics of the patients enrolled by diagnostic criteria for chronic HP are summarized in Table 1. Of the 56 chronic HP patients enrolled, 14 (25%) were admitted for acute deterioration of the disease (AE group) and other 42 cases had experienced no AE (NAE group). No significant differences in age or gender were observed between the AE and NAE groups, but AE was more likely to develop in ever smokers than in never smokers (p = 0.089). No significant differences between the two groups were found in the pulmonary function tests, in KL-6 and SP-D, biomarkers for interstitial pneumonia, or in HRCT findings on fibrosis and ground-glass scores. Histologically, the surgical lung specimens from the AE group had significantly more UIP patterns than the specimens from NAE group (p = 0.024). Patients received either corticosteroid (CS) alone, CS with immunosuppressants (IS) (cyclophosphamide [CPA] and cyclosporine A [CYA]), or no treatment. As no standardized or prospective treatment regimen was applied, the patients in the AE group received the combination therapy of CS and IS more frequently than they received no therapy or CS alone (p = 0.02). Meanwhile, a more fibrotic histological pattern (UIP > fNSIP > cNSIP) was correlated with a more intensive therapy (CS + IS > CS > no treatment) (r = 0.31, p = 0.026) in all patients. The median follow-up period was suggestive to be shorter in the AE group (27.5 months) than in the NAE group (53.0 months) (p = 0.055). Twenty-one of the 56 patients were lost to follow up because of transfer to other hospitals during the observation period, and the median follow-up period for these patients was 58 months. Twenty-three patients died during the follow-up period: 12 by AE, 6 by infection, and 5 by chronic progressive respiratory failure. The patients with AE tended to have fewer lymphocytes in their BALF (p = 0.085) than the patients in the NAE group (Table 2).

**Table 1:** Patient demographics at baseline†

**Characteristics**	**AE group (n = 14)**	**NAE group (n = 42)**	**p value**
Gender			0.350
Female	10	22	
Male	4	20	
Age, yr	65 (53–75)	64 (34–81)	0.557
Smoking			0.089
Never	4	23	
Ever	10	19	
VC,% predicted	68.6 (38.7-114.0)	80.7 (37.6-130.9)	0.279
PaO_2_, mm Hg	78.0 (49.8-94.1)	79.0 (47.6-98.0)	0.547
A-aDO_2_, mm Hg	18.1 (2.4-46.4)	17.4 (9.7-56.8)	0.622
KL-6, U/ml	1500 (867–5450)	1430 (319–8920)	0.737
SP-D, ng/ml	436.5(136–536)	236.5(49–934)	0.101
Fibrosis score	2.4 (0.8-2.9)	1.8 (0.0-3.8)	0.426
Ground-glass score	2.0 (0.6-4.0)	2.2 (1.2-4.8)	0.110
Histologic pattern			0.024*
UIP	12	19	
fNSIP	2	14	
cNSIP	0	9	
Treatment			0.020*
No treatment	0	9	
CS	4	19	
CS + I	10	10	
N/A	0	4	
Follow-up (months)	27.5 (5–128)	53.0 (2–162)	0.055

Definitions of abbreviation: *AE* acute exacerbations of chronic HP, *NAE* non-exacerbations of chronic HP, *VC* vital capacity, *PaO*_*2*_ partial pressure of carbon dioxide in arterial blood, *A-aDO*_*2*_ alveolar-arterial oxygen difference, *KL-6* Krebs on den Lungen-6, *SP-D* surfactant protein-D, *UIP* usual interstitial pneumonia, *fNSIP* fibrotic nonspecific interstitial pneumonia, *cNSIP* cellular nonspecific interstitial pneumonia, *CS* corticosteroid only, *I* immunosuppressant, *N/A* not available.

†Data are given as the median (range; minimum data-maximum data) or numbers.

*: p < 0.05.

**Table 2:** Profile of BAL at baseline†

**Variables**	**AE group (n = 11)**	**NAE group (n = 37)**	**p value**
Total cells, x10^6^	31.2 (12.7-58.7)	32.7 (5.6-114.3)	0.986
Macrophages, %	74.7 (33.7-93.0)	57.4 (14.0-98.0)	0.155
Lymphocytes, %	20.5 (5.0-62.2)	26.0 (2.0-86.0)	0.085
Neutrophils, %	1.6 (0.0-25.3)	1.6 (0.0-51.8)	0.484
Eosinophils, %	0.8 (0.0-20.5)	0.8 (0.0-18.8)	0.602
CD4/CD8 ratio	2.2 (0.5-19.5)	2.1 (0.2-15.8)	0.939

†Data are given as the median (range; minimum value-maximum value).

### Th1/Th2 chemokines and cytokines in serum and BALF at baseline

**Figure 1:**
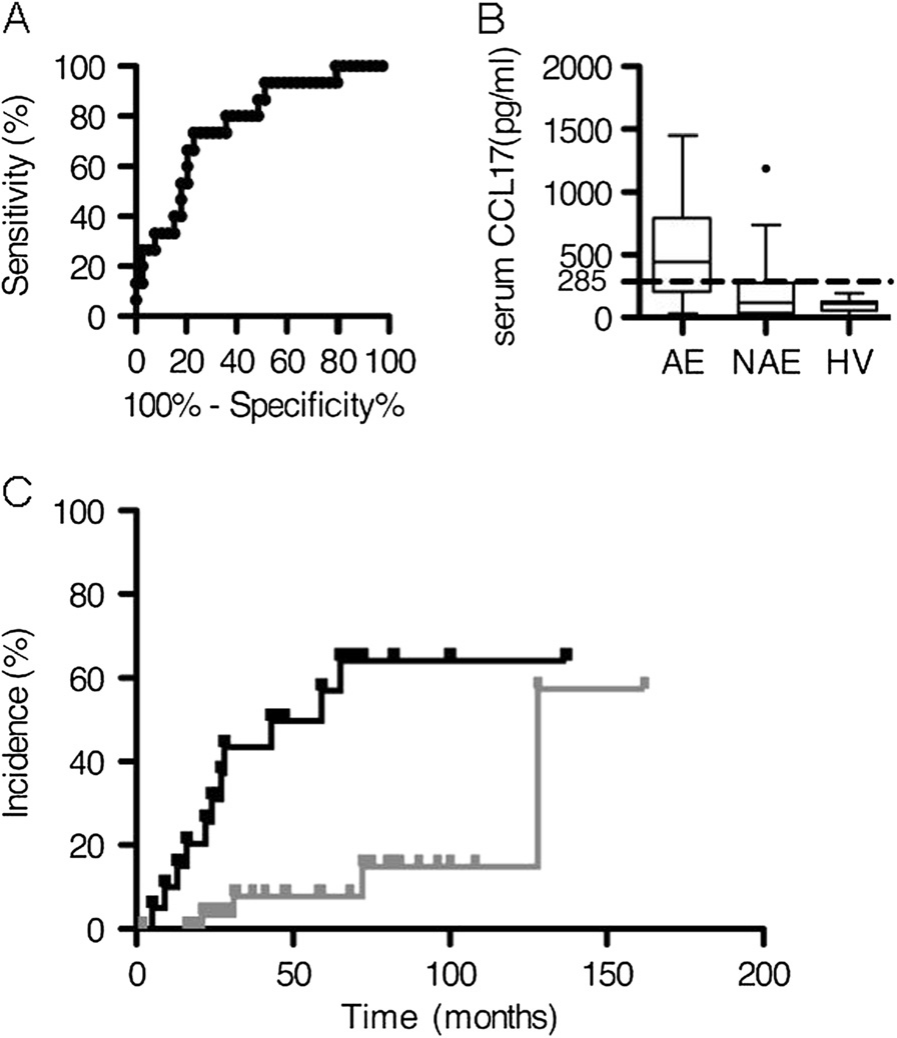
**Analysis of prognostic value of baseline serum CCL17. A**: Receiver operating curve analysis of baseline CCL17 levels using the first episode of AE as an endpoint (p = 0.02, AUC = 0.77). **B**: Box and whisker plots of baseline concentrations of CCL17 in AE and NAE groups, and healthy volunteers (HV) as a reference. The *dotted line* represents the cutoff value for predicting AE. **C**: The incidence of acute exacerbations grouped by baseline serum concentrations. The *black line* represents the chronic HP patients with baseline serum CCL17 concentrations above 285 pg/ml. The *gray line* represents the chronic HP patients with baseline serum CCL17 concentrations under 285 pg/ml (log rank test, p = 0.0006).

Of the serum markers, the levels of CCL17 and IL-4 were significantly higher in the AE group than in the NAE group (AE vs. NAE: CCL17, 450.8 [29.4-1450] vs. 132.9 [5.3-1189], p = 0.003; IL-4, 14.0 [0.0-73.7] vs. 13.0 [0.0-43.9], p = 0.036). There were no differences in CCL17 levels between the NAE group and healthy volunteers (NAE vs. HV: 116.7[5.3-1189] vs. 112.3[0.0-193.6], p = 0.468) (Figure 1B). In the BALF examinations, the levels of TGF-β·were significantly higher in the NAE group (387.3 [0.0-1782]) than in the AE group (0.0 [0.0-796.1]) (p = 0.042) (Table 3). However, only one AE sample demonstrated detectable levels of TGF-β in BALF.

**Table 3:** Chemokines and cytokines in serum and BALF†

**Variables**	**AE group (n = 14)**	**NAE group (n = 42)**	**p value**
Serum CXCL10 (pg/ml)	53.9 (18.9-210.1)	74.9 (2.8-1493.8)	0.291
Serum CCL17 (pg/ml)	450.8 (29.4-1450)	132.9 (5.3-1189)	0.003**
Serum IL-4 (pg/ml)	14.0 (0.0-73.7)	13.0 (0.0-43.9)	0.036*
Serum IL-13 (pg/ml)	148.3 (0.0-974.7)	144.6 (0.0-550.7)	0.622
Serum TGF-β (ng/ml)	23.4 (4.5-299.5)	18.8 (1.7-172.2)	0.272
Serum IFN-γ (pg/ml)	ND	ND	
Variables	AE group (n = 11)	NAE group (n = 37)	p value
BALF CXCL10 (pg/ml)	59.0 (24.1-160.4)	83.7 (9.1-733.7)	0.624
BALF CCL17 (pg/ml)	8.1 (0.0-38.9)	5.9 (0.0-32.9)	0.233
BALF IL-4 (pg/ml)	ND	ND	
BALF IL-13 (pg/ml)	31.4 (0.0-241.2)	68.7 (0.0-290.4)	0.851
BALF TGF-β (ng/ml)	0.0 (0.0-796.1)	387.3 (0.0-1782)	0.042*
BALF IFN-γ (pg/ml)	ND	ND	

Definitions of abbreviation: *ND* not detected, *NS* not significant.

†Data are given as the median (range; minimum value-maximum value).

*: p < 0.05, **: p < 0.01.

### Prognostic value of baseline serum CCL17 concentrations for predicting the first episode of AE

ROC analysis was performed to test whether baseline serum CCL17 concentrations were predictive of the first episode of AE. Baseline serum CCL17 concentrations had a significant relationship with the first episode of AE (p = 0.002 and AUC = 0.77, Figure 1A). The cut-off point for serum CCL17 concentrations was determined to be 285 pg/ml (sensitivity, 0.77; specificity, 0.73, Figure 1B). Accordingly, we divided the patients into two groups; a higher-CCL17 group (n = 20, baseline CCL17 concentrations ≥ 285 pg/ml) and a lower-CCL17 group (n = 36, baseline CCL17 concentrations < 285 pg/ml). The incidences of AE for the two groups were plotted using a Kaplan-Meier survival curve (Figure 1C). The 1-year and 2-year incidences of AE were 14.3% and 30.1% in the higher-CCL17 group, versus 0.0% and 3.3% in the lower-CCL17 group. AE was significantly more frequent in the higher-CCL17 group than in the lower CCL17 group (Figure 1C, log-rank test, p = 0.0006).

### Factors predictive of the first episode of AE evaluated by Cox proportional hazard models

We explored how robustly chemokines and cytokines predicted an upcoming episode of AE. In the univariate model, a baseline serum CCL17 above 285 pg/ml (binary response for CCL17) was associated with an increased risk of AE (hazard ratio [HR], 5.42; 95% CI, 1.69-17.30; p = 0.004), but no such association was found in any of other chemokines or cytokines in serum (Table 4). Multivariate analyses were performed on two models with the same set of variables, namely, CCL17, CXCL10, IL-4, IL-13, TGF-β, age, gender, positive smoking history and VC%. The response for CCL17 was binary in Model 1 and continuous in Model 2. None of the baseline demographic or pulmonary function variables (e.g. age, gender, positive smoking history, VC%) were significant in either model. In Model 1, CCL17 (binary response) had a p value of 0.002 and an HR of 72.00 with a 95%CI (5.03-1030.23). This was highly significant. In Model 2, CCL17 (continuous response) had a p value of 0.038 and an HR of 1.0023 with a 95%CI (1.0001-1.0045).

**Table 4:** Univariate Cox proportional hazard model evaluating the incidence of AE

	**Hazard ratio**	**95%CI**		**p value**
Age, yr	1.04	0.98	1.11	0.200
Gender (male = 1)	2.59	0.81	8.34	0.110
Positive smoking history	1.81	0.20	16.20	0.600
VC, % predicted	0.99	0.96	1.01	0.220
CXCL10	0.99	0.98	1.00	0.130
CCL17 (binary)	5.42	1.69	17.30	0.004**
CCL17 (continous)	1.00	1.00	1.00	0.003**
IL-13	1.00	1.00	1.00	0.980
IL-4	1.02	1.00	1.05	0.068
TGF-β	1.00	1.00	1.00	0.072

Definition of abbreviation: CI; confidence interval.

Cutoff point of binary CCL17: 285 pg/ml.

**: p < 0.01.

### Immunohistological localization of CCL17 and CCR4 in surgical lung specimens

**Figure 2:**
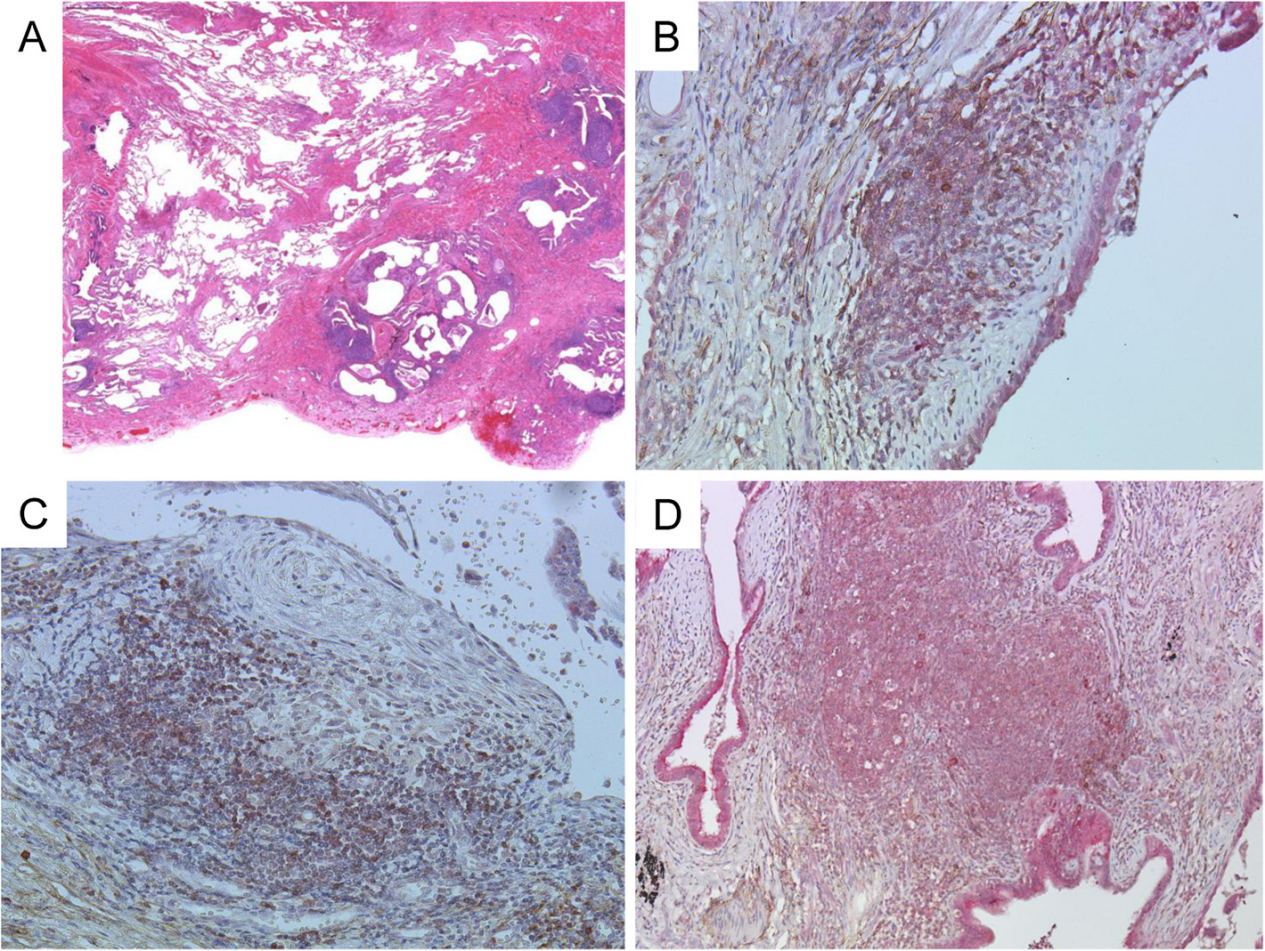
**Representative picture and immunohistochemistry of CCL17 and CCR4 in the surgical lung biopsy specimen from AE patient. A**: Low-power microscopic view, haematoxylin-eosin stain. Magnification, 1.5×. Bar = 2000 μm. **B**, **C** and **D**: Immunohistochemistry (IHC) of CCL17 and CCR4. **B**, fibrosing area and fibroblastic foci, 200×; **C**, fibroblastic foci, 200×; and **D**, lymphoid cluster, 100x. For IHC, red represents CCL17 staining and brown represents CCR4 staining.

In the surgical biopsy specimens at the time of diagnosis in 14 AE patients, a UIP pattern was frequently observed; 12 patients had a UIP pattern and 2 patients had a NSIP pattern (Table 1). A low-power microscopic view (Figure 2A) showed the UIP pattern, including the subpleural fibrosis with alternating normal alveoli, fibroblastic foci and honeycomb changes. Lymphocyte aggregations were observed mainly in the fibrotic lesions; however, no granuloma and no NSIP patterns were revealed.

**Figure 3:**
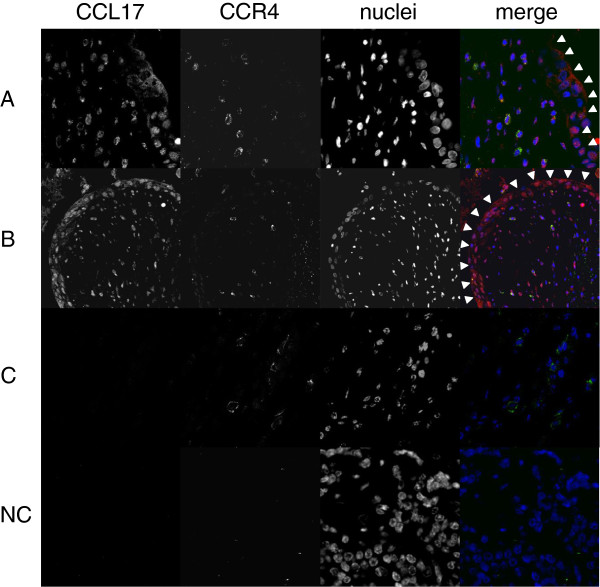
**Immunofluorescent of CCL17 and CCR4 in the surgical lung biopsy specimen of AE patients.** Locations of critical structures are as follows: **A**, fibrosing areas; **B**, fibroblastic foci; and **C**, lymphoid clusters. In merged images, red represents CCL17 staining, green represents CCR4 staining, and blue represents nuclear staining. Epithelial cells are outlined with arrowhead. Magnification, 1200x. NC: negative control.

**Figure 4:**
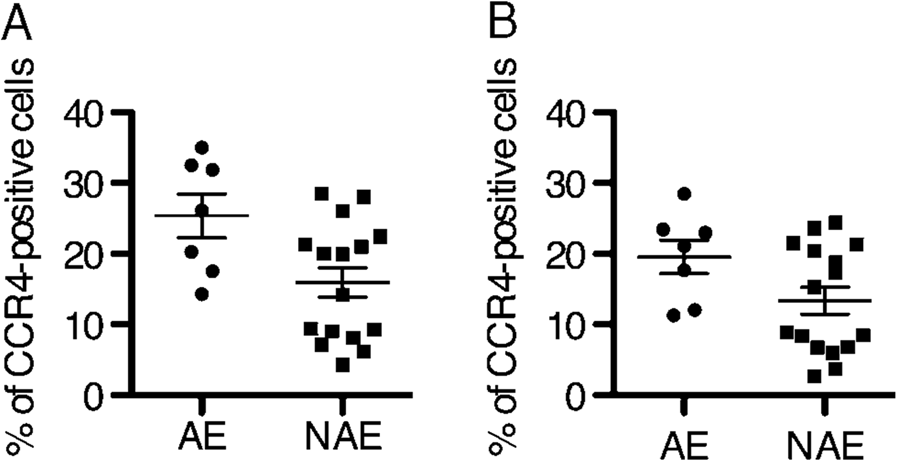
**Comparison of CCR4-positive cells in the lungs between AE patients and NAE patients.** Comparison of CCR4-positive cells in fibrosing areas (**A**, p = 0.035) or in lymphoid clusters (**B**, p = 0.088) in surgical lung biopsy specimens taken from the AE group compared with the same in specimens taken from the NAE group.

We evaluated the role of CCL17 in the lungs by performing immunohistochemistry for CCL17 and CCR4 on surgical lung specimens (7 patients from the AE group and 16 patients from the NAE group). Intense CCL17 immunostaining was found in epithelial cells (Figure 2B, 2C, 2D, and Additional file 1: Figure S1A), while CCL17-positive cells were rarely observed in lymphoid clusters (Figure 2D, 3C, and Additional file 1: Figure S1B). CCR4-positive cells can be observed more often in fibrotic lesion than in lymphoid clusters, and CCR4-positive cells are clustered together in proximity to CCL17-positive cells in fibrotic area and fibroblastic foci but not in lymphoid clusters (Figure 3A, 3B, 3C, Additional file 1: Figure S1A, and S1B). The CCL17-positive cells were morphologically identified as bronchiolar epithelial cells and hyperplastic epithelial cells. The percentage of CCR4-positive cells in fibrosing areas was significantly higher in specimens from the AE group than in specimens from the NAE group (Figure 4A, p = 0.035). Meanwhile, we found suggestive difference between the AE and NAE groups in the percentage of CCR4-positive cells in lymphoid clusters (Figure 4B, p = 0.088). The correlation between the serum CCL17 concentration and the percentage of CCR4-positive cells was positive by Spearman’s rank correlation test (vs. CCR4+ cells in lymphoid clusters, r = 0.520, p = 0.005, CCR4+ cells in fibrosing area, r = 0.415, p = 0.032).

### Comparison of serum CCL17 and CCR4-positive cells between at baseline and during the episode of AE

**Figure 5:**
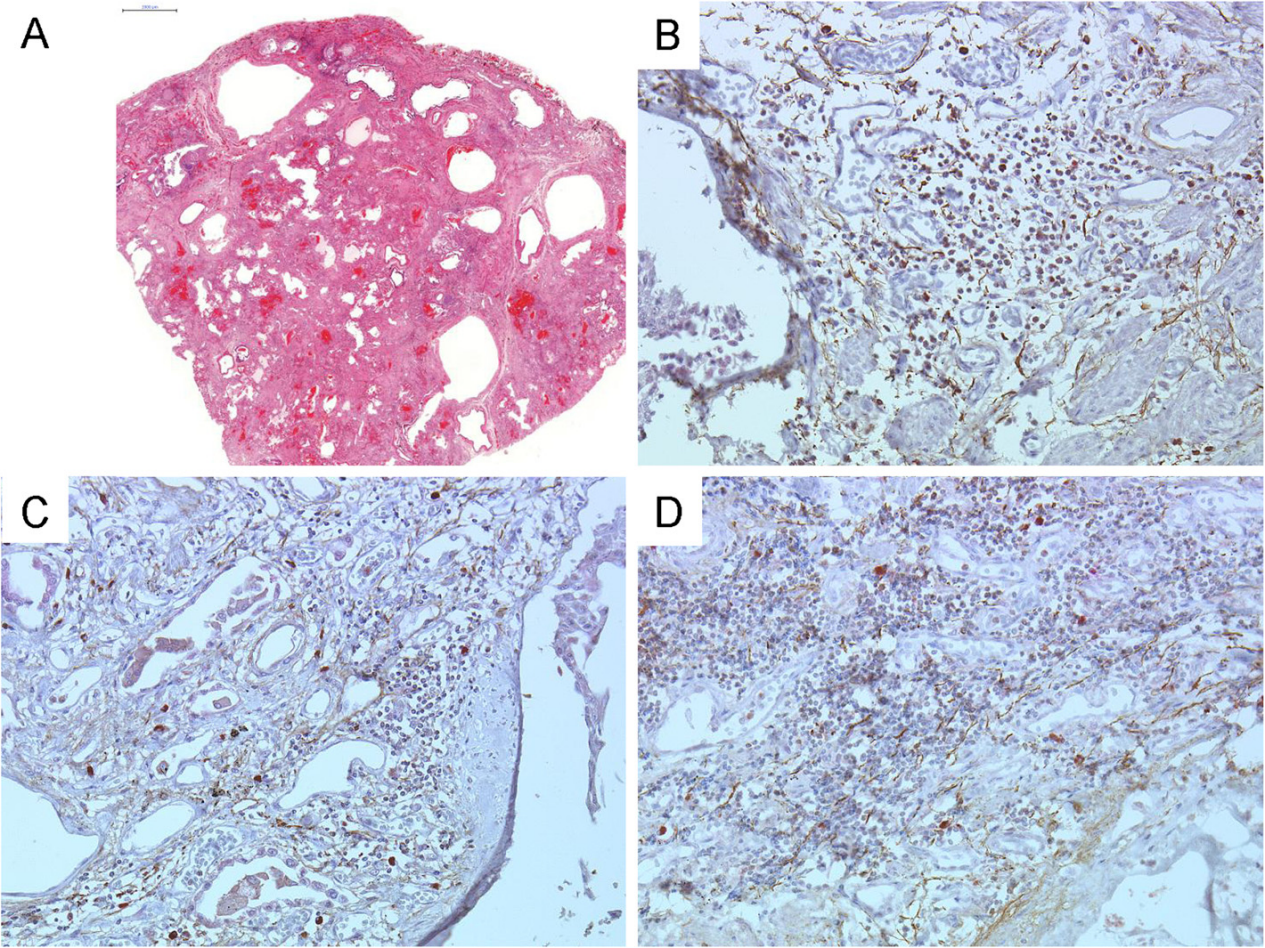
**Representative picture of autopsy lung specimen form AE patient.** The images of the autopsy lung from the same patient as Figure 2. **A**: Low-power microscopic view, haematoxylin-eosin stain. Magnification, 1×. Bar = 2000 μm. **B**, **C** and D: Immunohistochemistry (IHC) of CCL17 and CCR4. **B**, fibrosing areas, 200×; **C**, fibroblastic foci, 200×; and **D**, lymphoid clusters, 200×. For IHC, red represents CCL17 staining and brown represents CCR4 staining.

The representative image, Figure 5A is an image of the autopsy lung from the same patient as shown in Figure 2. The image revealed a UIP pattern, dense subpleural atelectatic fibrosis and honeycomb changes. The lymphocyte accumulations were loose compared with the surgical biopsy specimens as shown in Figure 2A.

**Figure 6:**
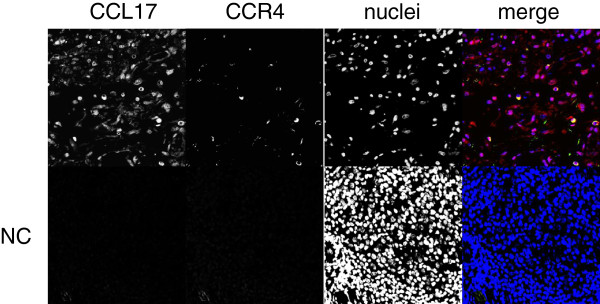
**Immunofluorescent of CCL17 and CCR4 in the autopsy lungs of AE patients.** In merged images, red represents CCL17 staining, green represents CCR4 staining, and blue represents nuclear staining. Magnification, 600×. NC: negative control.

**Figure 7:**
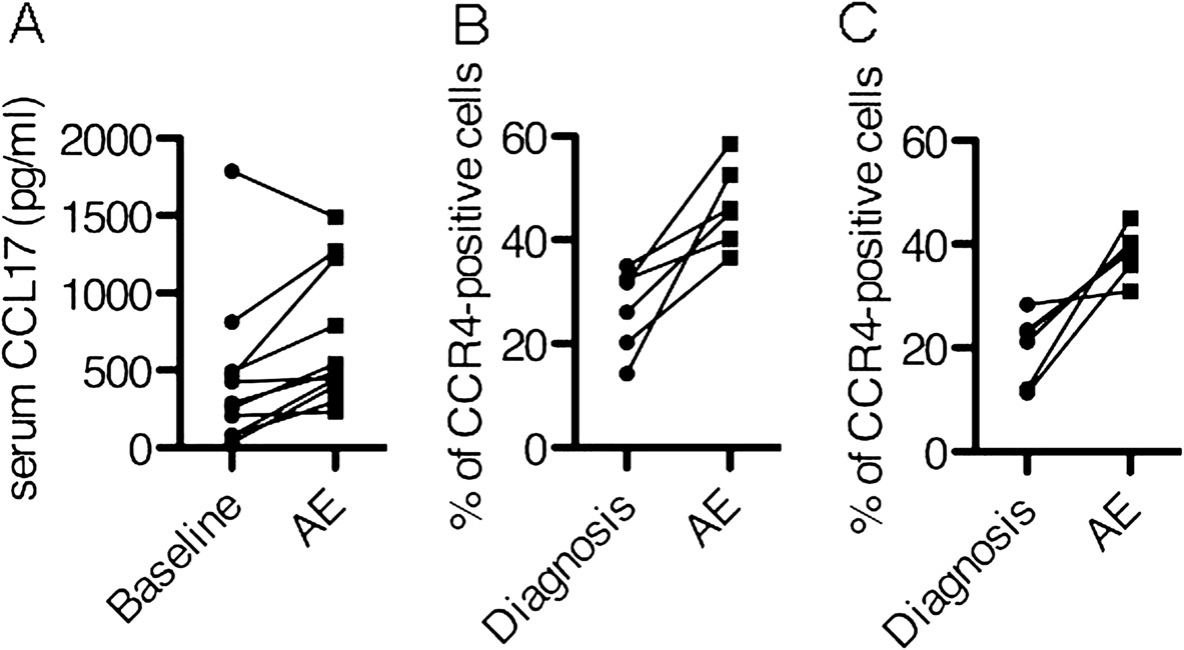
**Comparison of Serum CCL17 and CCR4-positive cells between baseline and AE. A**: Comparison of serum CCL17 concentrations between baseline and AE (p = 0.01). **B** and **C**: Comparison of CCR4-positive cells in fibrosing areas (**B**, p = 0.031) and in lymphoid clusters (**C**, p = 0.031) in surgical lung biopsy specimens taken at the time of diagnosis compared with the same in specimens taken during an episode of AE.

In autopsy specimens from cases who died during AE episodes, CCL17 was expressed in epithelial cells and fibroblasts (Figure 5B, C, and D). Many more CCR4-positive cells were observed in both the fibrotic area and the lymphocyte accumulation area compared with the surgical lung specimen in Figure 2A. CCR4- and CCL17-double-positive cells were present in fibrosing areas from the autopsy specimens with AE (indicated by white arrowheads in Figure 6). Paired samples were measured at baseline and during AE in 11 patients from the AE group. Serum CCL17 concentrations were significantly increased during AE compared to the baseline (p = 0.01) (Figure 7A). Immunohistochemistry for CCR4 was performed on paired lung specimens at the time of diagnosis and during an AE episode in 6 patients. The percentage of CCR4-positive cells was significantly higher during AE than at baseline in lymphoid clusters and in fibrosing areas (Figure 7B and C; p = 0.031 and p = 0.031, respectively).

## Discussion

This study assessed potential associations between Th1/Th2 biomarkers and AE episodes in patients with chronic HP. We found that the baseline CCL17 concentration was an independent predictor of the first episode of AE. Furthermore, CCR4-postitive cells were significantly increased in lung specimens from the AE group compared to those from the NAE group at the time of diagnosis, and CCL17 and CCR4 levels were significantly increased during AE compared to baseline.

By analyzing chemokines/cytokines in serum and BALF, we clearly showed that a cut-off point of 285 pg/ml in serum CCL17 was an independent indicator of the incidence of AE in chronic HP patients that we studied. A binary classification test with a sensitivity of 0.77 and specificity of 0.73 is not sufficient, as this fails to identify about 30% of the patients at risk (Figure 1B). We can, however, identify about 70% of the AE candidate at the time of diagnosis. While no markers strongly predictive of AE are yet available, vigorous investigations by numerous researchers have identified ST2 [25], α-defensins [26] and circulating fibrocytes [27] in peripheral blood as biomarkers for AE. In an earlier study we found that the baseline serum CCL17 is higher in patients with UIP patterns than in patients with NSIP pattern [8], and findings from the present study identified the UIP pattern as a good predictor of AE (Table 1). Accordingly, we performed a ROC curve analysis in patients with UIP patterns to clarify the effect of the underlying histological patterns. As it turns out, the baseline serum CCL17 is significantly associated with AE in UIP patients, and the strength of the association is similar to that in patients including all histological patterns (UIP pattern, AUC = 0.74, p = 0.035 vs. all histological patterns, AUC = 0.77, p = 0.002) (Figure 1A). From the viewpoint of safety, surgical lung biopsy to identify the histological patterns is invasive and elevates the risk of AE. Serum biomarkers, on the other hand, can be sampled easily and safely.

CCL17 is a Th2 chemokine mainly produced by epithelial cells during allergic pulmonary inflammation [12,28]. CCL17 is established to be involved in three Th2-dominant diseases: bronchial asthma, atopic dermatitis, and eosinophilic pneumonia [29]. Increased concentrations of CCL17 are initially found in the BAL fluid of IPF patients, which suggests that CCL17 is involved in the pathogenesis of IPF via the recruitment of Th2 cells [12,30]. Regarding chronic HP, we showed that elevated levels of CCL17 may contribute to the development of UIP histological patterns [8]. Increased serum CCL17 levels at baseline were predictive of AE in chronic HP in the present study. The elevated CCL17 in this population may be partly linked to a continuous and unrecognized exposure to low-dose avian antigens through sources such as feather duvets, pet birds in the office, pigeons raised by neighbors, flocks of pigeons or wild birds in parks, shrines and railway stations. A detailed environmental survey by site visits confirmed that all of the AE patients investigated had been unanimously exposed to these sources, whereas most of the NAE patients visited had not (unpublished data). Continued antigen exposure of this type could skew a patient’s Th1/Th2 balance towards Th2. In another paper we reported that longer exposure to bird-antigen increased Th2 cytokine such as IL-13 [16]. The suggestive effects of smoking might also be responsible for the elevated CCL17 in our subjects. Ever smokers were suggestively frequent in the AE group (p = 0.089). Long-term smoking per se was found to decrease the expression of Th1 cytokines in a mouse model of BFL [31], and to favor the development of Th2 responses [32]. CCL17 production by BALF cells increases after smoking in acute eosinophilic pneumonia patients [29], and elevated *CCL17* mRNA levels were observed in a murine model of cigarette smoke-induced airway inflammation [33]. Compared to the NAE group, the AE group had a higher proportion of smokers and a significant higher proportion of ex-smokers. This may have been due to involuntary cessation of smoking before the onset of AE in the AE group because of severely impaired pulmonary function tests. Virus infection may be another cause of elevated CCL17. We excluded infections in all patients by evaluating sputum cultures, blood cultures, and serological findings such as endotoxin, β-D glucan and cytomegalovirus (CMV). However, we did not consider other viral species. Wootton *et al.* have identified an unexpected virus (torque teno virus: TTV) that was associated with 33% of acute exacerbations, although they did not suggest that viral infection was a major cause of acute exacerbation in IPF patients. It is possible that the presence of TTV represents a consequence of lung inflammation rather than the cause of AE [34]. In the study of viral infections, respiratory syncytial virus (RSV) is of great interest. RSV is a ubiquitous virus that preferentially infects airway epithelial cells, causing asthma exacerbations and severe disease in immunocompromised hosts. Monick *et al*. showed that RSV and Th2- biased environments could synergistically induced CCL17 and recruit Th2 cells [35].

CCR4 is a CCL17 receptor preferentially expressed on Th2 cells [11]. Patients with IPF have significantly higher CCR4 expression on BAL CD4 T cells compared to patients with other interstitial lung diseases such as sarcoidosis [36]. Our group has shown that CCL17- and CCR4-positive cells contribute to the pathogenesis of UIP patterns [8]. Further, a study using Cox regression hazard models revealed higher mortality risks in cases with UIP patterns detected by HRCT [37]. These data prompted us to investigate whether chemokines and their receptors play a significant role in the pathogenesis of AE in chronic HP with a UIP pattern. Intense staining with CCL17 was seen in fibroblastic foci and in bronchiolar epithelial cells adjacent to fibrosing areas in patients with AE, and there was a correlation between baseline serum CCL17 levels and the accumulation of CCR4-positive cells in surgical lung biopsies. Meanwhile, we found that serum CCL17 levels and CCR4-positive cells were both increased during AE versus the baseline levels (Figure 5). These data suggest that CCL17 may be involved in fibrogenesis and the development of AE via the recruitment of CCR4-positive cells into the lesions. In the present study we are unable to show the higher CCL17 levels in association with the CCR4-positive cells of the lungs. As the protein in BALF is 100 times less concentrated than that of alveolar lining fluid [38], it was difficult to detect an elevation in the alveolar CCL17 concentration. The levels of BALF CCL17· were considerably low in the AE and NAE group, 8.1 (0.0-38.9) pg/ml and 5.9 (0.0-32.9) pg/ml, respectively, and lower than the detectable range in 5 of 11 AE patients and in 19 of 37 NAE patients. Miyazaki *et al*. also showed that the serum CCL17 concentration of patients with sarcoidosis and IPF are higher than in healthy volunteers despite having undetectable concentrations of BALF CCL17 [39].

This study had several limitations. First, the study was retrospective. While our results demonstrated a possibility that CCL17 may be predictive of AE, we will need to conduct a prospective study to certify the results. In addition, there was a significant referral bias in the study population, as our hospital is a clinical center for chronic HP.

## Conclusions

In conclusion, higher serum concentrations of baseline CCL17 may be a promising predictive marker of AE in patients with chronic HP, and CCL17 may contribute to the pathology of AE by inducing the accumulation of CCR4-positive lymphocytes in the lungs.

## Abbreviations

AE: Acute Exacerbations; BALF: Bronchoalveolar Lavage Fluid; BFL: Bird Fanciers’ Lung; CCL: C-C chemokine Ligand; CCR: C-C chemokine Receptor; CPA: Cyclophosphamide; CS: Corticosteroids; CXCL: C-X-C chemokine Ligand; CXCR: C-X-C chemokine Receptor; CYA: Cyclosporine A; HP: Hypersensitivity Pneumonitis; IPF: Idiopathic Pulmonary Fibrosis; IFN: Interferon; IL: Interleukin; IS: Immunosuppressant; KL: Krebs on den Lungen; MMP: Matrix Metalloproteinase; NAE: Non-AE; NSIP: Non-Specific Interstitial Pneumonia; ROC: Receiver Operating Characteristic; SP: Surfactant Protein; TGF: Transforming Growth Factor; UIP: Usual Interstitial Pneumonia; VC: Vital Capacity.

## Competing interests

None of the authors have declared that they have conflict of interest in the authorship or publication of this contribution.

## Authors’ contribution

YM contributed to the conception and design of the study; collecting, analyzing, and interpreting the data; and drafting the manuscript. KU contributed to analyzing and interpreting the data. TT contributed to collecting, analyzing, and interpreting the data. TA contributed to interpreting the histopathology of surgical biopsies. TT contributed to interpreting the histopathology of surgical biopsies. MT contributed to analyzing, and interpreting the data. NI contributed to the conception and design of the study; and drafting the manuscript. YY contributed to the conception and design of the study; and drafting the manuscript. All authors read and approved the final manuscript.

## Supplementary Material

Additional file 1: Figure S1 High-power images of immunohistochemistry for CCL17 and CCR4 in the surgical lung biopsy specimen from the AE patient. **A**: High-power image of Figure 2B, fibrosing area and fibroblastic foci. Magnification, 400×. **B**: High-power image of Figure 2D, lymphoid cluster. Magnification, 400×. Red represents CCL17 staining, and CCL17-positive cells are indicated by arrows. Brown represents CCR4 staining, and CCR4-positive cells are indicated by arrowheads. Click here for file
