# Supplementary figures and images for: Higher serum CCL17 may be a promising predictor of acute exacerbations in chronic hypersensitivity pneumonitis
Source: Respir Res. 2013 May 25;14(1):57. doi: 10.1186/1465-9921-14-57 (PMC3665443; doi:10.1186/1465-9921-14-57)

## Slide 1
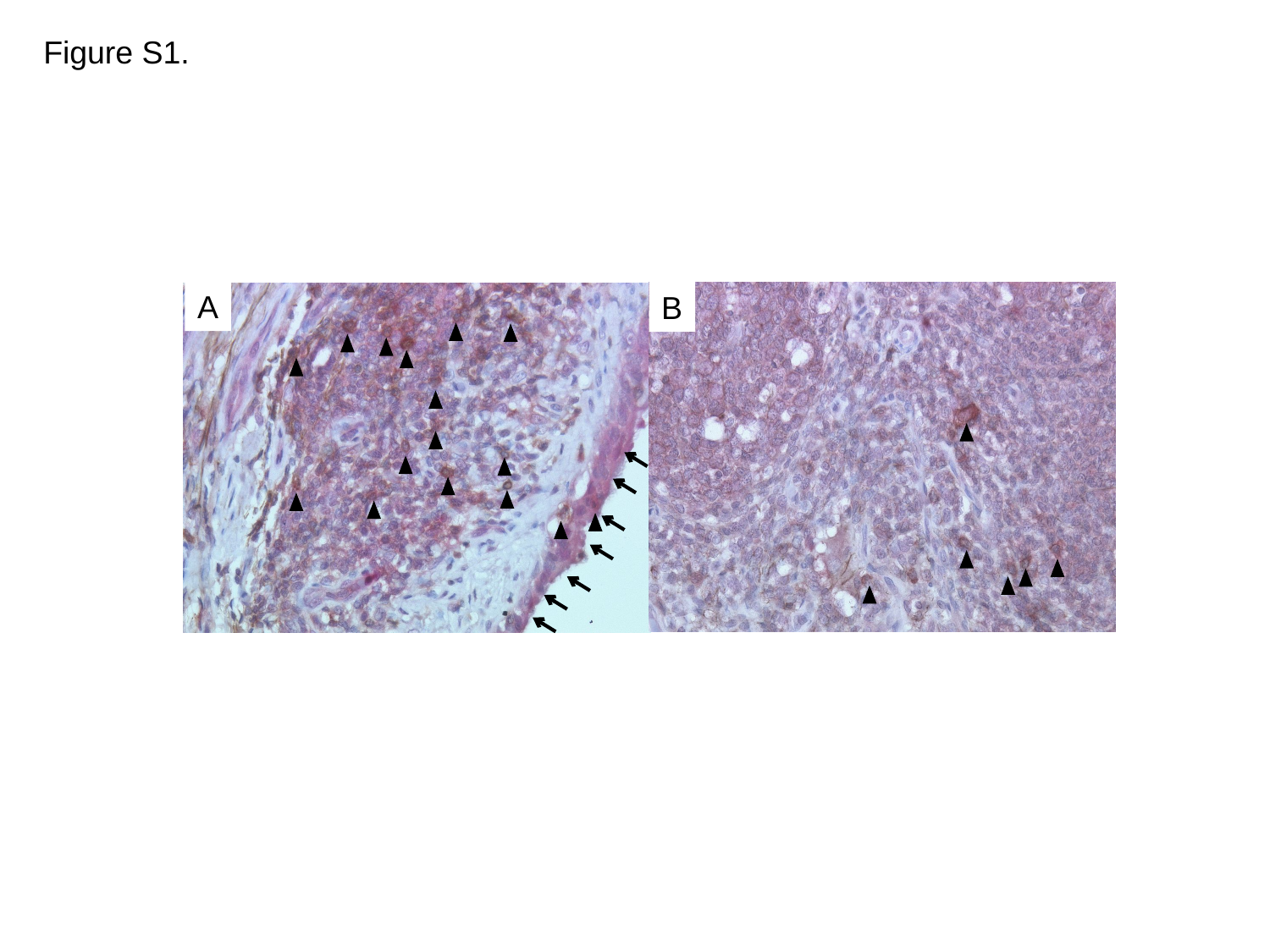

Figure S1.
A
B

Supplement: Additional file 1: Figure S1 — High-power images of immunohistochemistry for CCL17 and CCR4 in the surgical lung biopsy specimen from the AE patient. A: High-power image of Figure 2B, fibrosing area and fibroblastic foci. Magnification, 400×. B: High-power image of Figure 2D, lymphoid cluster. Magnification, 400×. Red represents CCL17 staining, and CCL17-positive cells are indicated by arrows. Brown represents CCR4 staining, and CCR4-positive cells are indicated by arrowheads. [file 1465-9921-14-57-S1.ppt]
